# Comparison of Surgical Outcomes of Laparoscopic Glue and Laparoscopic Suture Hernioplasty in Pediatric Female Inguinal Hernia

**DOI:** 10.3390/children9050724

**Published:** 2022-05-15

**Authors:** In Geol Ho, Kyong Ihn, Ho Jong Jeon, Yonghyun Na, Dongeun Lee, Seok Joo Han

**Affiliations:** 1 Department of Pediatric Surgery, Severance Children’s Hospital, Yonsei University College of Medicine, Seoul 08308, Korea; hnjklop@yuhs.ac (I.G.H.); pedsik@yuhs.ac (K.I.); gdijhj@yuhs.ac (H.J.J.); solar730@yuhs.ac (D.L.); 2 Department of Surgery, Division of Pediatric Surgery, Korea University Guro Hospital, Korea University College of Medicine, Seoul 08308, Korea; herian0830@gmail.com

**Keywords:** pediatric inguinal hernia, laparoscopic glue hernioplasty, laparoscopic suture hernioplasty, surgical outcomes

## Abstract

This study aimed to report the surgical outcomes of laparoscopic glue hernioplasty (LGH) compared with conventional laparoscopic suture hernioplasty (LSH) in pediatric female inguinal hernia repair. We retrospectively analyzed 465 female pediatric patients who underwent laparoscopic inguinal hernia repair between January 2013 and December 2020. LGH and LSH were performed in 95 and 370 cases, respectively. Surgical outcomes (length of hospital stay, operative time, complications, and recurrences) were compared between the LGH and LSH groups. We found that the operation times for bilateral hernia repair were shorter in the LGH group (LGH: 35.5 ± 8.2 min, LSH: 45.2 ± 11.6 min; *p* < 0.001). No significant differences in complications or recurrences were observed between the two groups during the follow-up period. Our findings suggest that LGH is a feasible and easily applied surgical technique for the treatment of pediatric female inguinal hernia.

## 1. Introduction

Inguinal hernia is one of the most common pediatric surgical diseases [1]. With modern advancements in anesthesia and surgical instrument refinements, laparoscopic hernioplasty has become a popular choice for pediatric surgeries [2,3]. According to a recent meta-analysis of evidence from the last 10 years, the recurrence rates for laparoscopic and open inguinal hernia repair are similar (odds ratio [OR] 1.05, *p* = 0.66) [4]. The recurrence rates for inguinal hernia repair are lower in children than in adults, ranging from 0.5 to 4% [5], while those following laparoscopic repair range from 0.7 to 4.5% [6].

However, there is still an ongoing debate about the best approach and the advantages and disadvantages of intracorporeal [7,8] and extracorporeal methods [9,10]. Both surgical methods involve suturing for the closure of the internal inguinal ring. Unfortunately, the correct placing of the internal inguinal suturing is difficult and requires laparoscopic surgical skills. The intracorporeal method typically requires a surgeon who is highly skilled in laparoscopy to suture the inguinal ring and tie the knots. Recently, percutaneous internal ring suturing (PIRS), which represents a kind of extracorporeal method, has been developed [11,12,13]. As conversion rates have been reported to reach a plateau after surgeons have performed at least 30 procedures [14,15], PIRS has been spotlighted by some pediatric surgeons as a technically easier, safe, and effective approach for pediatric hernia repair. However, extracorporeal methods require an extra incision, and some procedures can only be performed with specially designed instruments [10]. In addition, adverse events associated with extraperitoneal knotting, such as infection, pain, blood vessel injury, and a palpable thread knot around the inguinal area of the abdominal wall, might occur with extracorporeal methods [16,17].

To overcome these limitations, using glue as a tissue adhesive has been proposed as an alternative to conventional laparoscopic inguinal hernioplasty [18]. In 1835, Velpeau observed an inguinal hernia that was cured by the accidental introduction of iodine into the inguinal canal during the injection of a hydrocele [19]. Since then, glue has been used internally and externally on the human body [20,21,22]. Previously, the effectiveness and safety of laparoscopic glue hernioplasty (LGH) were evaluated in a series of animal studies [18,23,24]. Here, we report our single-institution experience, which provides evidence that the LGH technique is effective in treating inguinal hernia in pediatric female patients [25]. However, so far, there are no reports on how LGH results compare to conventional laparoscopic suture hernioplasty (LSH). Thus, this study aimed to compare the surgical outcomes of LGH with those of conventional LSH in the treatment of pediatric female inguinal hernias.

## 2. Materials and Methods

### 2.1. Patients

We retrospectively analyzed the medical records of patients with inguinal hernias who were treated at Severance Children’s Hospital between January 2013 and December 2020. Inclusion criteria were (i) aged 0 to 18 years and (ii) female sex. Exclusion criteria were (i) older than 18 years of age and (ii) a combination surgery including another procedure. The choice of surgical method was surgeon-dependent, and determined by the surgeon’s experience, beliefs, and confidence in their skills to perform a certain technique. Ultimately, 465 patients were included in the study. LGH was performed in 95 cases and LSH in 370; accordingly, the patients were divided into an LGH and an LSH groups. This study was reviewed and approved by the Institutional Review Board of Yonsei University Health System, Severance Hospital (approval date: 27 January 2021; approval No. 4-2020-1368). The requirement for informed consent was waived because of the retrospective design of the study.

### 2.2. Outcome Measurements

The primary outcome of the study was to evaluate surgical outcomes regarding operation time, complication, and recurrence. The secondary outcome was to compare the surgical outcomes of the two groups and confirm the novel surgical method–(LGH)’s safety and efficiency. A further follow-up evaluation was performed through a telephonic survey.

### 2.3. Statistical Analysis

All data were analyzed using IBM^®^ SPSS^®^ Statistics version 25 (IBM, Armonk, NY, USA). Two-sample *t*-tests, chi-squared tests, and the Mann–Whitney U test were used to analyze the data; *p*-values < 0.05 were considered statistically significant.

### 2.4. Surgical Techniques

**Figure 1:**
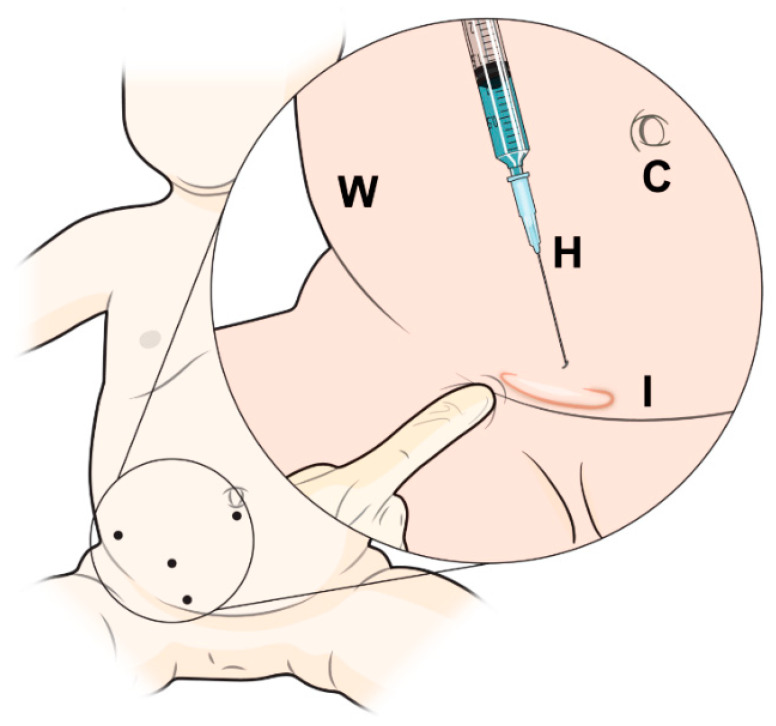
Schematic image of laparoscopic glue hernioplasty. **C**: Camera, **W**: Abdomen wall, **H**: Histoacryl^®^, **I**: Inguinal ring.

**Figure 2:**
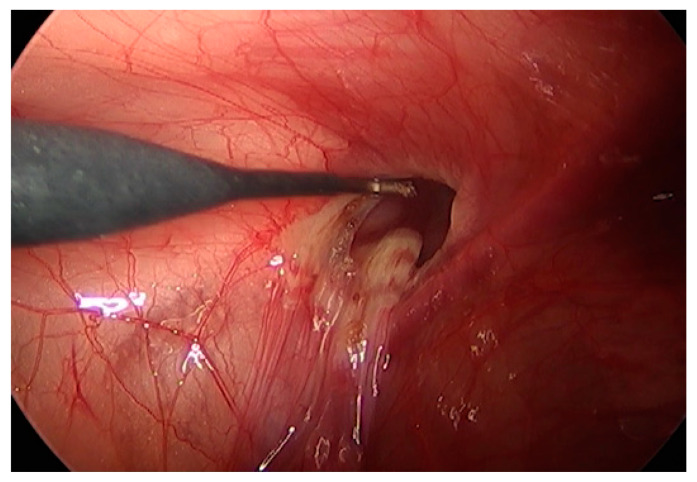
The mesothelial layer surrounding the internal ring was electrically cauterized.

**Figure 3:**
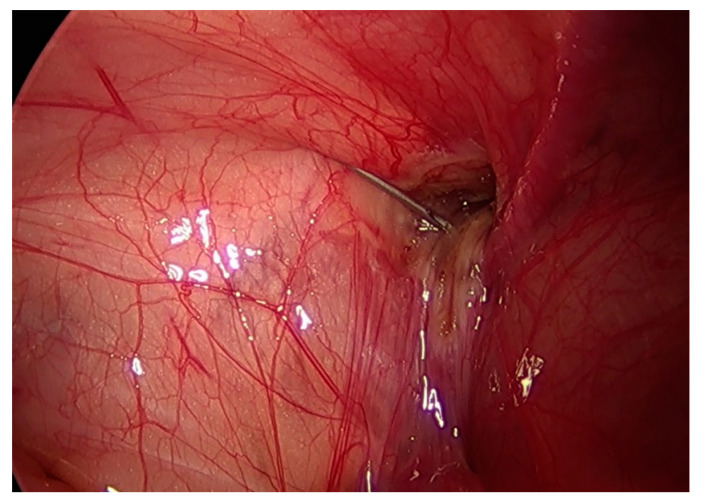
Histoacryl^®^ was injected and a long 25-gauge needle was used to close the internal inguinal ring.

We adopted the previously published techniques of LGH [25] and LSH [8]. All procedures were performed while the patients were under general anesthesia. After the trocars were introduced, a pneumoperitoneum was created with a resulting intraabdominal pressure of 5–12 mmHg. For the LSH procedure, three trocars were used: a 5 mm optical trocar was inserted through the umbilicus, and two 3 mm trocars were inserted lateral to the rectus abdominis muscle, slightly below the level of the umbilicus. The mesothelial layer surrounding the internal inguinal ring was electrically cauterized, the peritoneum was circumferentially divided at the level of the internal inguinal ring, and the hernia ring was ligated using purse-string sutures with a 3-0 non-absorbable suture (3-0 Ethibond Excel, Ethicon, Somerville, NJ, USA) (Online Resource S1: laparoscopic suture hernioplasty). For LGH, two trocars were used: a 5 mm camera port was inserted through the umbilicus and a 3 mm instrument working port was inserted lateral to the rectus abdominis muscle. The internal inguinal ring was divided in the same way as during LSH; following electrical cauterization, the internal inguinal ring was narrowed by applying external pressure through finger pushing (Figure 1 and Figure 2). A long, 25-gauge needle attached to a 1 mL syringe filled with N-butyl-2-cyanoacrylate (b-can; Histoacryl^®^; B. Braun, Melungeon, Germany) was then inserted into the peritoneal cavity, 2–3 cm above the targeted internal inguinal ring. The injection of 0.5–1 mL of Histoacryl^®^ was generally sufficient to close the internal inguinal ring (Figure 3). The peritoneum was held in place until the Histoacryl^®^ hardened, after approximately 4–5 s. The closure of the internal ring was then confirmed by probing with the laparoscopic instrument (Online Resource S2: laparoscopic glue hernioplasty).

## 3. Results

### 3.1. Demographics

The clinical characteristics of the patients are shown in Table 1.

The median age at the time of surgery was 45 months (range: 1–138 months) in the LGH group and 48 months (range: 0–188 months) in the LSH group (*p* = 0.063). The mean body weight at the time of surgery was 15.6 ± 7.19 kg in the LGH group and 15.5 ± 8.9 kg in the LSH group (*p* = 0.065). There were no statistically significant differences in age or weight at the time of surgery between the two groups. Lateralization in the LGH group was 25 (right) and 33 (left), compared to 110 (right) and 82 (left) in the LSH group. There were 37 LGH and 178 LSH bilateral hernia repairs. There were no significant differences in the type of surgery performed between the two groups (p = 0.058 and p = 0.11).

### 3.2. Operative Time

The summarized surgical outcomes are shown in Table 2.

With respect to the mean operative time for the repair of unilateral inguinal hernias, there was no significant difference between the LGH and LSH groups (LGH: 32.2 ± 15.4 min, LSH: 37.5 ± 12.4 min; *p* = 0.26). However, the mean operative time for the repair of bilateral inguinal hernias was shorter for the LGH than for the LSH group (LGH: 35.5 ± 8.2 min, LSH: 45.2 ± 11.6 min; *p* < 0.001).

### 3.3. Length of Hospital Stay

Most patients were discharged on the day of their surgery or 1 day after the surgery. There was no significant difference between the two groups in this regard (LGH: 1.06 ± 1.05 days, LSH: 0.93 ± 0.99 days; *p* = 0.412).

### 3.4. Follow-Up and Recurrence

There were two recurrence cases in the LGH group and none in the LSH group during the follow-up (LGH: 124.7 ± 61.2 months, LSH: 102.8 ± 61.1 months; *p* = 0.433). However, there was no significant difference between the two groups (*p* = 0.062).

### 3.5. Postoperative Complications

In the LGH group, postoperative wound infection in the umbilical trocar insertion site occurred in three patients. In the LSH group, postoperative wound infection occurred in four patients and postoperative ileus occurred in two patients. The cause of the ileus was paralytic ileus after surgery, and the patients recovered after conservative treatment. There was no significant difference between the two groups (*p* = 0.332).

## 4. Discussion

In this study, we assessed the outcomes of a novel surgical technique (LGH) and compared them to those of the conventional surgical method (LSH) for pediatric inguinal hernia repair in female patients. We found shorter operation times for LGH bilateral hernioplasty and good cosmetic effects compared to conventional LSH.

Minimally invasive surgery has been the major revolutionary change in many conventional surgical procedures, and has resulted in a remarkable reduction in morbidity [26,27,28]. After the introduction of laparoscopic hernia repair, the paradigm of pediatric inguinal hernia treatment has changed to laparoscopic surgery as an alternative to the existing open inguinal hernia repair approach [2,3,29]. Various laparoscopic surgical methods have been developed that can be classified into two major approaches: intracorporeal [7,8] and extracorporeal [15,30] methods.

Advantages of laparoscopic inguinal hernia repair have been reported, such as the ability to evaluate the contralateral side, avoid excess trauma to the spermatic cord structures, iatrogenic cryptorchidism, testicular atrophy, as well as less postoperative pain [31]. However, over the last decade, the laparoscopic technique for inguinal hernia repair in children has not undergone major technical advances [3,13,32]. The existing laparoscopic surgical technique has some disadvantages regarding the closing of the internal inguinal ring. Intracorporeal methods require advanced laparoscopic skills, and are technically challenging and time consuming [3]. Recently, percutaneous internal ring suturing (PIRS) procedures, which represent a kind of extracorporeal method, have been developed; these techniques are technically easier to implement than intracorporeal methods, and lead to lower recurrence rates and shorter operation times [11]. In addition, they have been reported as safe and effective not only for young children, but also for young adolescent patients [33], and have, thus, been spotlighted by some pediatric surgeons. However, extracorporeal methods require an extra incision, and some can only be conducted with specially designed instruments. In addition, adverse events associated with extra peritoneal knotting, such as infection, pain, and a palpable thread knot around the inguinal area of the abdominal wall, have been reported for extracorporeal methods [16,17]. These issues have limited the widespread use of such methods in pediatric surgical practice. Ideally, an advanced technique of laparoscopic inguinal hernia repair should be simple and easy to adopt, and not come with the above limitations.

To overcome these challenges, the use of glue in combination with a laparoscopic surgical technique has been developed and published as an attractive, easy, and reproducible technique [18,19,24,25]. Various studies have reported on surgical glue injection into the human body, and there has been much discussion about the safety and efficacy of this approach [21,22,34].

Cyanoacrylates (CNAs) were developed in the 1960s and 1970s. Short-chain CNAs were found to be toxic to human tissue and to cause tissue necrosis. Conversely, long-chain CNAs, such as b-CNA and 0-CNA, are degradable and can be used in humans, without adverse effects [35]. In the pediatric surgery field, glue has been used to reduce the risk of postoperative complications, especially in cases of parenchymal resection or vascular anastomosis [36,37]. Glue has also been applied in pediatric laparoscopic surgery as a good means of controlling bleeding, particularly in cases of spleen or liver bleeding, and is a valid method for completing and securing sutures in cases of organ perforation [34].

One important characteristic of b-CNA (Histoacryl^®^) is that when it comes into contact with a liquid, it is polymerized through an exothermic reaction to form a strong bond. However, it produces heat during the polymerization process, releases toxic products that inhibit cell growth, and is not completely biodegradable. Therefore, potential concerns related to Histoacryl^®^ use include the possibility of a foreign body response and tissue damage from the exothermic reaction during polymerization [38]. Adverse effects related to these characteristics of b-CNA, such as mild chronic inflammation and obstruction due to tissue adhesion and tissue toxicity, have been reported during long-term follow-ups after intraperitoneal interventions [39,40].

For this reason, and considering that not much has been reported on the stability of glue, our study only included female patients to avoid potential damage to the vas deferens. In previous studies, Kato and Ayman reported that the use of glue was effective in the repair of inguinal hernia, with no recurrence on long-term follow-up, no damage to spermatic cord structures, and no clear impact on fertility [18,41]. Similarly, in our study, the glue was partially absorbed after LGH, which was confirmed by a second surgery due to ovarian torsion, where the glue injection site remained completely closed, without any intraperitoneal organ adhesion [42]. In contrast, Miyano et al. reported concerns on the increased risk of intestinal obstruction by adhesive syndrome, hernia recurrence, postoperative hydrocele, and long-term carcinogenesis [24]. Recently, Maria et al. published a comparative study on the laparoscopic injection of tissue adhesives and standard laparoscopic suture inguinal hernia repair in a male rabbit model. The authors describe several critical issues that are associated with a higher incidence of postoperative complications and a significant decrease in testicular maturity in tissue adhesive models [43].

As an alternative, we are focusing on bioinspired tissue adhesives going forward. These adhesives were developed recently [44] and are receiving attention as potential biomedical materials in various clinical fields [45,46]. Mussel adhesive proteins (MAPs) have been considered as a potential bio-adhesive, due to their unique features [47,48]. MAPs are able to maintain their adhesion in a wet environment, and such water-immiscible mussel adhesives have potential diverse biotechnological applications, such as cell and tissue adhesives. They also have the advantage of being biodegradable, with less toxicity [49,50]. More advanced MAPs are currently being developed, based on recombinant DNA and bioengineering technology. According to recent research based on animal experiments, regenerative bio-glue leads to better results and reduces foreign body reactions in the treatment of vesico-vaginal fistula, compared with existing glue and surgical suture repair [51]. Therefore, MAPs can be used as medical adhesives as they are harmless to the human body. In the future, through animal experiments, we will further research the safety and efficacy of bioinspired adhesives, specifically MAPs, in the treatment of pediatric inguinal hernias.

Our study provides important findings regarding the use of LGH. We found significantly shorter operative times for LGH than for LSH in bilateral hernia repair, and no significant difference in surgical complications or recurrence rates. Additionally, LGH requires only two laparoscopic ports, which makes it superior to conventional LSH, not only in terms of its easy adoption, but also its better cosmetic outcomes. Similar to our findings, a previous study reported that patients who underwent laparoscopic inguinal hernia repair exhibited lower levels of pain and inflammatory stress responses than those treated with an open approach [52]. Therefore, in our study, we assume that LGH is the less invasive and easier operation, and it reduces surgery-related stress for the patient.

These advantages are considered to provide a significant benefit to surgeons who are not experts in laparoscopic surgery. In low- and middle-income countries, pediatric inguinal hernia is still a major cause of morbidity and mortality, yet elective surgical treatment remains largely unavailable [53,54]. The development of such an easy surgical method is, thus, regarded to be a great help in the treatment of pediatric inguinal hernia in developing countries.

The results of this study show that, compared with conventional LSH, LGH is technically easy, reproducible, has a shorter operating time, and does not require advanced laparoscopic skills; therefore, it can be easily applied in the treatment of pediatric female inguinal hernia. The limitations of our study include the absence of male patients, the fact that it was a single-center study, and the retrospective study design, which makes it difficult to extrapolate our findings to the entire pediatric population. Moreover, the safety of the glue is still controversial. Further research regarding the stability and safety of bioinspired adhesives in the treatment of pediatric inguinal hernia is warranted.

## Figures and Tables

**Table 1:** Characteristics of patients.

Characteristics	Total	LGH ^a^	LSH ^b^	*p*-Value
**Cases (female sex)**	465	95	370	
**Lateralization**				
Unilateral	250	58	192	
Right	(135)	(25)	(110)	0.058
Left	(115)	(33)	(82)	
Bilateral	215	37	178	0.11
**Median age (months) at operation**		45 (1–138)	48 (0–188)	0.063
**Mean body weight (kg) at operation**		15.6 ± 7.19	15.5 ± 8.9	0.065

^a^ LGH: laparoscopic glue hernioplasty; ^b^ LSH: laparoscopic suture hernioplasty.

**Table 2:** Surgical outcomes.

	LGH (95)	LSH (370)	*p*-Value
**Operative time (min)**			
**Unilateral hernia repair**	32.2 ± 15.4	37.5 ± 12.4	0.26
**Bilateral hernia repair**	35.5 ± 8.2	45.2 ± 11.6	<0.001
**Length of hospital stay (days)**	1.06 ± 1.05	0.93 ± 0.99	0.412
**Length of follow-up period (months)**	124.7 ± 61.2	102.8 ± 61.1	0.433
**Recurrence**	2	0	0.062
**Postoperative wound infection**	3	4	0.332
**Postoperative ileus**	0	2	

## Data Availability

The datasets generated and/or analyzed during the current study are available from the corresponding author on reasonable request.

## Supplementary Materials

The following are available online at https://doi.org/10.5281/zenodo.6475697: Online Resource S1: laparoscopic suture hernioplasty; Online Resource S2: laparoscopic glue hernioplasty.
